# Early life body size and puberty markers as predictors of breast cancer risk later in life: A neural network analysis

**DOI:** 10.1371/journal.pone.0296835

**Published:** 2024-02-09

**Authors:** Sara M. S. Svendsen, Dorthe C. Pedersen, Britt W. Jensen, Julie Aarestrup, Lene Mellemkjær, Lise G. Bjerregaard, Jennifer L. Baker

**Affiliations:** 1 Center for Clinical Research and Prevention, Copenhagen University Hospital—Bispebjerg and Frederiksberg, Copenhagen, Denmark; 2 Danish Cancer Institute, Copenhagen, Denmark; Taipei Medical University, TAIWAN

## Abstract

**Background:**

The early life factors of birthweight, child weight, height, body mass index (BMI) and pubertal timing are associated with risks of breast cancer. However, the predictive value of these factors in relation to breast cancer is largely unknown. Therefore, using a machine learning approach, we examined whether birthweight, childhood weights, heights, BMIs, and pubertal timing individually and in combination were predictive of breast cancer.

**Methods:**

We used information on birthweight, childhood height and weight, and pubertal timing assessed by the onset of the growth spurt (OGS) from 164,216 girls born 1930–1996 from the Copenhagen School Health Records Register. Of these, 10,002 women were diagnosed with breast cancer during 1977–2019 according to a nationwide breast cancer database. We developed a feed-forward neural network, which was trained and tested on early life body size measures individually and in various combinations. Evaluation metrics were examined to identify the best performing model.

**Results:**

The highest area under the receiver operating curve (AUC) was achieved in a model that included birthweight, childhood heights, weights and age at OGS (AUC = 0.600). A model based on childhood heights and weights had a comparable AUC value (AUC = 0.598), whereas a model including only childhood heights had the lowest AUC value (AUC = 0.572). The sensitivity of the models ranged from 0.698 to 0.760 while the precision ranged from 0.071 to 0.076.

**Conclusion:**

We found that the best performing network was based on birthweight, childhood weights, heights and age at OGS as the input features. Nonetheless, this performance was only slightly better than the model including childhood heights and weights. Further, although the performance of our networks was relatively low, it was similar to those from previous studies including well-established risk factors. As such, our results suggest that childhood body size may add additional value to breast cancer prediction models.

## Introduction

Among women, breast cancer is the most commonly diagnosed cancer form worldwide and it is the leading cause of death from cancer [1]. As such, it is important to identify women at high risk for breast cancer as early as possible. To aid this, different breast cancer risk prediction tools have been developed to improve and streamline screening approaches [2]. Two of the most widely used breast cancer risk assessment tools are the Gail model/ the Breast Cancer Risk Assessment Tool (BCRAT) and the Rosner-Colditz model [2], which include well-established risk factors such as ages at menarche and menopause, parity, use of hormone replacement therapy, and a family history of breast cancer [3, 4]. In addition, the Rosner-Colditz model includes information on average adult BMI before and after menopause as well as adult height [5].

Since the development of these prediction tools, a large body of research has shown that body size early in life relates to risks of breast cancer [6]. However, the associations are complex as birthweight and childhood height are positively associated with risks of breast cancer [7, 8], whereas childhood body mass index (BMI), weight, self-reported body shape and age at peak growth are inversely associated with risks of breast cancer [7, 9–11]. Recently, a study examined the effect of additionally including adolescent somatotypes (a proxy of childhood adiposity) in the Rosner-Colditz model, which significantly improved the model performance [12], and thus, highlighting the potential importance of early life body size in relation to prediction of breast cancer. Nevertheless, the importance of including other measures of early life body size together with measures of childhood adiposity in relation to prediction of breast cancer remains largely unknown. As such, a machine learning approach may be a suitable tool to understand the predictive power of these measures and their complex associations with breast cancer, since the method does not focus on inference, but learns from the data and finds predictive patterns. Therefore, we used a neural network to examine whether birthweight, childhood heights, weights, BMIs, and pubertal timing individually and in combination were predictive of breast cancer.

## Materials and methods

### Data material

Information on early life body size and puberty markers were obtained from the Copenhagen School Health Records Register (CSHRR), which currently includes 200,978 girls born during 1930–1996 [13]. In the municipality of Copenhagen, virtually every schoolchild underwent regular health examinations performed by school physicians and nurses. Height and weight were measured and for children born from 1936, birthweight was reported by the parents at the first health examination using either the child’s health booklet or recall. From the height measurements, age at onset of the pubertal growth spurt (OGS) and age at peak height velocity (PHV) were derived as described in detail previously [14]. The ages at OGS and PHV were estimated for girls born from 1930–1969, which is the period with a sufficient number of height measurements for its determination. Missing values for all variables were imputed over 10 iterations with the multivariate imputer from sklearn, where a regressor was fit at each step, with one feature as output and the rest of the features as input. The method is similar to multiple imputation by chained equations but returns only a single imputation [15].

A personal identification number from the Danish Civil Registration System has been issued to all Danish residents alive or born after 1968 [16]. These numbers were recorded in the register for girls still attending school in 1968 and were retrieved for those who left school before this time [13]. We excluded girls without this number (n = 21,856), with the most common reasons for not having one being emigration or death prior to 1968. Moreover, we excluded women with less than three values of height, weight, or BMI from ages 7–13 years (n = 14,906) leaving a total of 164,216 girls for analysis.

Via the personal identification number, girls in the CSHRR were linked to the nationwide Danish Breast Cancer Group (DBCG) database, which contains information on women diagnosed with a first primary breast cancer since 1977 [17]. Breast cancer status was obtained through 2019.

The Danish Data Protection Agency approved the project and the data-linkage. According to Danish law, ethical approval is not required for purely register based studies of pre-existing personal data.

### Feature selection

Height, weight and BMI at ages 7–13 years as well as birthweight and ages at OGS and PHV were included as continuous variables. A preliminary feature selection showed that age at OGS had a higher power than age at PHV. Thus, we only included age at OGS in the subsequent networks.

Five separate networks were trained on (1) BMIs, (2) weights, (3) heights, (4) both heights and weights, and (5) birthweight, heights, weights and age at OGS. These networks were chosen on the basis of a previous study we conducted on the same data-resource, where we reported consistent associations of childhood BMIs and heights with breast cancer risks, but not with birthweight and the markers of puberty [18]. Women were divided in two classes; women diagnosed with a breast cancer in the DBCG database constituted the breast cancer class and the remaining women constituted the non-cancer class.

### Neural network architecture

To predict which girls developed breast cancer later in life, we implemented a network with five layers consisting of an input layer, three hidden layers and an output layer. After each hidden layer, rectified linear unit (Relu) activation function was applied [19]. After the output layer, sigmoid was used as the activation function to convert the outputs to a number between 0 and 1.

### Loss function

Cross entropy was the loss function with Adam as the optimizer [20]. Class weights were assigned to account for the imbalance in the classes of non-case and cancer. The class weights were used for weighting the loss function penalizing misclassification of the minority class, in our analyses this was the breast cancer class. The weight for the class *i* is defined by:

$$clas{s\;weight}_{i}=\frac{N}{2*N_{i}}$$

where *N* is the total number of women in the training data and *N_i_* is the number of women belonging to class *i*.

### Training and testing

**Fig 1:**
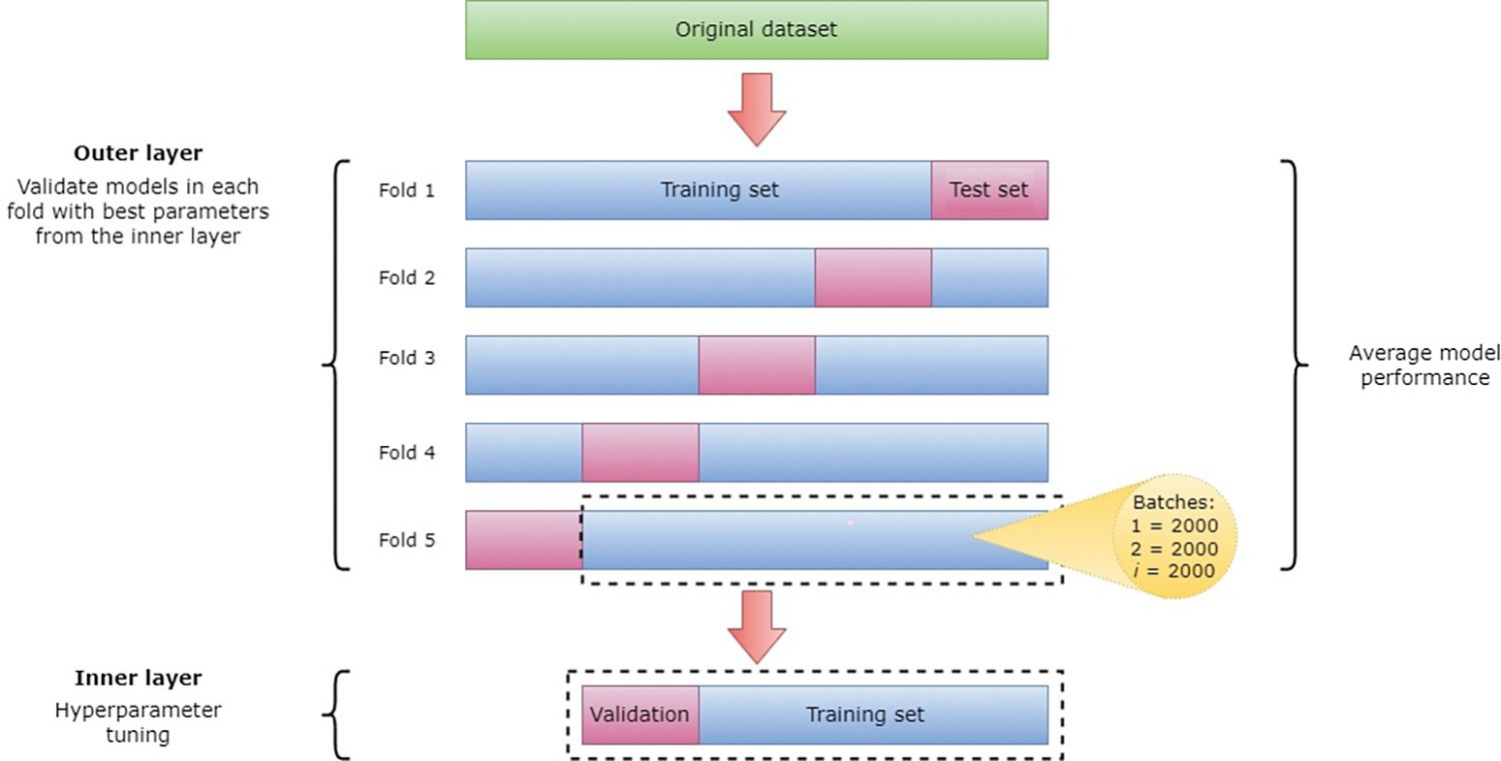
Overview of the five-fold nested cross validation process.

The networks were trained and tested in a five-fold nested cross validation. In each of the five data splits, 80% of the data were used for training and 20% were used for testing. The distribution of classes from the entire dataset was maintained using stratified K-fold. The training data was standardized in each outer layer of the cross validation, and the test data was then standardized with the standardization parameters obtained from the training set. The networks were trained on batches of 2000 women at the time to ensure that both classes were present in most batches. In the inner layer, 20% of the training set was used as validation when tuning the hyperparameters. After the hyperparameters were determined, the network was retrained over 100 epochs with the best hyperparameters, and the evaluation metrics were calculated on the test set. The process is illustrated in Fig 1. The sub-samples of individuals used for training, validation or testing respectively, were the same for all five models.

### Hyperparameters optimization

The number of neurons in the hidden layers and the learning rate was tuned as hyperparameters with random search. The number of hidden neurons was allowed to range from eight to 20 as an even number, and the learning rate was either 0.01 or 0.001. The tuning was based on minimizing the loss function, with 10 random combinations of hyperparameters tested. The model was trained over 50 epochs for each combination of the hyperparameters. A seed was set for the search algorithm, the weight initialization and data split for reproducibility and to enable a fairer comparison of networks.

### Evaluation metrics

The network performance was evaluated by the area under the receiver operating characteristic curve (AUC), accuracy, specificity, sensitivity and precision. The metrics are defined as:

$$Sensitivity=\frac{True\;Positives}{True\;Positives+False\;Negatives}$$ (1)

$$Specificity=\frac{True\;Negatives}{True\;Negatives+False\;Positives}$$ (2)

$$Precision=\frac{True\;Positives}{True\;Positives+False\;Positives}$$ (3)

$$Accuracy=\frac{True\;Negatives+True\;Positives}{True\;Negatives+False\;Negatives+True\;Positives+False\;Positives}$$ (4)

The performance of the network is reported as the mean and standard deviation of the evaluation metrics from the cross-validation folds. We further evaluated the five different networks using logistic regression and compared the performance metrics from these models with those from the neural networks.

### Software

The neural network was developed in the open-source platform TensorFlow. The implementation and data handling were performed with Keras and sklearn libraries in Python version 3.7.

## Results

Among the 164,216 women included in the study, 10,002 were diagnosed with a primary breast cancer between 1977 and 2019. Women with breast cancer had a median age of 60.0 years (25^th^ and 75^th^ percentiles: 51.4 and 67.6 years) at diagnosis. Summary characteristics of the input variables among women with and without breast cancer are shown in Table 1. Percentages of imputed values (range: 1.5–54.4%) are shown in S1 Table.

**Table 1:** Characteristics of the study population by breast cancer status.

	Breast cancer status	
Input variable	Yes (n = 10,002)	No (n = 154,214)
Birthweight, kg	3.3 (3.0, 3.5)	3.3 (3.0, 3.5)
Height, m		
Age 7 years	1.22 (1.18, 1.25)	1.22 (1.19, 1.25)
Age 8 years	1.27 (1.23, 1.30)	1.27 (1.24, 1.31)
Age 9 years	1.32 (1.28, 1.36)	1.32 (1.29, 1.36)
Age 10 years	1.37 (1.33, 1.41)	1.38 (1.34, 1.41)
Age 11 years	1.43 (1.38, 1.47)	1.43 (1.39, 1.47)
Age 12 years	1.49 (1.44, 1.54)	1.50 (1.45, 1.54)
Age 13 years	1.55 (1.51, 1.60)	1.56 (1.52, 1.60)
Weight, kg		
Age 7 years	22.3 (20.5, 24.1)	22.8 (20.9, 24.7)
Age 8 years	24.8 (22.8, 27.0)	25.4 (23.1, 27.7)
Age 9 years	27.6 (25.3, 30.2)	28.4 (25.7, 31.0)
Age 10 years	30.5 (27.8, 33.6)	31.6 (28.4, 34.3)
Age 11 years	34.0 (30.7, 37.8)	35.4 (31.4, 38.6)
Age 12 years	38.6 (34.5, 43.0)	40.2 (35.4, 44.0)
Age 13 years	44.1 (39.4, 48.6)	45.5 (40.6, 49.4)
BMI, kg/m^2^		
Age 7 years	15.1 (14.4, 15.9)	15.4 (14.5, 16.2)
Age 8 years	15.5 (14.7, 16.3)	15.7 (14.8, 16.6)
Age 9 years	15.8 (15.0, 16.8)	16.1 (15.2, 17.1)
Age 10 years	16.2 (15.3, 17.3)	16.5 (15.5, 17.6)
Age 11 years	16.7 (15.7, 17.8)	17.0 (15.9, 18.2)
Age 12 years	17.3 (16.2, 18.5)	17.7 (16.4, 18.9)
Age 13 years	18.2 (16.9, 19.5)	18.6 (17.2, 19.8)
Age at OGS, years	10.2 (9.6, 10.9)	10.2 (9.5, 10.8)

Data are presented as median (25^th^, 75^th^ percentile)

Abbreviations: BMI, body mass index; OGS, onset of the growth spurt

**Fig 2:**
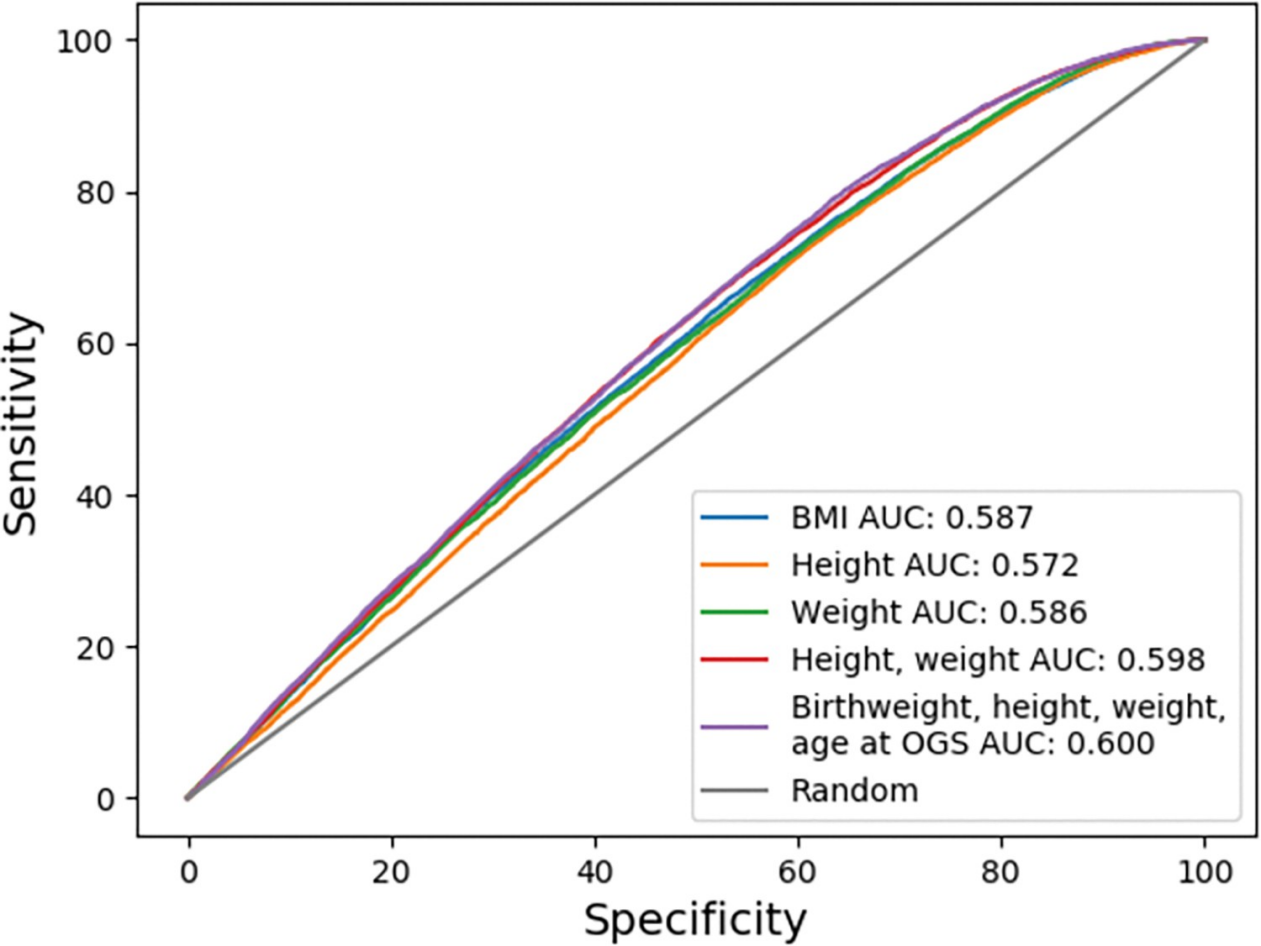
ROC curves for the neural networks with early life body measures predicting breast cancer. Abbreviations: AUC, area under the curve; BMI, body mass index; OGS, onset of the growth spurt; ROC, Reciever operating characteristics.

The performance of the networks that were trained and tested with the different sets of early life measures as the inputs can be seen in Table 2. Overall, the network including birthweight, heights, weights, and age at OGS had the highest AUC of 0.600, but it varied little from the reduced model that only included heights and weights (AUC = 0.598). The lowest performing network included only heights (AUC = 0.572) (Table 2). Nevertheless, the AUC achieved by the five models varied slightly, which is depicted in Fig 2. In general, the sensitivity was higher than the specificity in all the networks, but none of the networks predicted the risk of breast cancer with an accuracy above 0.463 (Table 2). Further, the precision was low for all networks ranging from 0.071 to 0.076 (Table 2).

**Table 2:** Performance metrics for the neural networks predicting breast cancer*^,^**.

	Network performance metrics				
Input variables	AUC	Sensitivity	Specificity	Accuracy	Precision
BMI	0.587 (0.005)	0.706 (0.047)	0.418 (0.042)	0.436 (0.037)	0.073 (0.001)
Height	0.572 (0.005)	0.760 (0.010)	0.354 (0.018)	0.378 (0.017)	0.071 (0.001)
Weight	0.586 (0.005)	0.742 (0.042)	0.380 (0.044)	0.402 (0.039)	0.072 (0.001)
Height, weight	0.598 (0.003)	0.698 (0.042)	0.447 (0.039)	0.463 (0.034)	0.076 (0.001)
Birthweight, height, weight, age at OGS	0.600 (0.007)	0.726 (0.026)	0.422 (0.028)	0.441 (0.024)	0.075 (0.001)

* Data are presented as mean and standard deviation of the cross-validation folds

** The input variables BMI, height, and weight includes values at each age between 7 and 13 years

Abbreviations: AUC, area under the curve; BMI, body mass index; OGS, onset of the growth spurt

**Fig 3:**
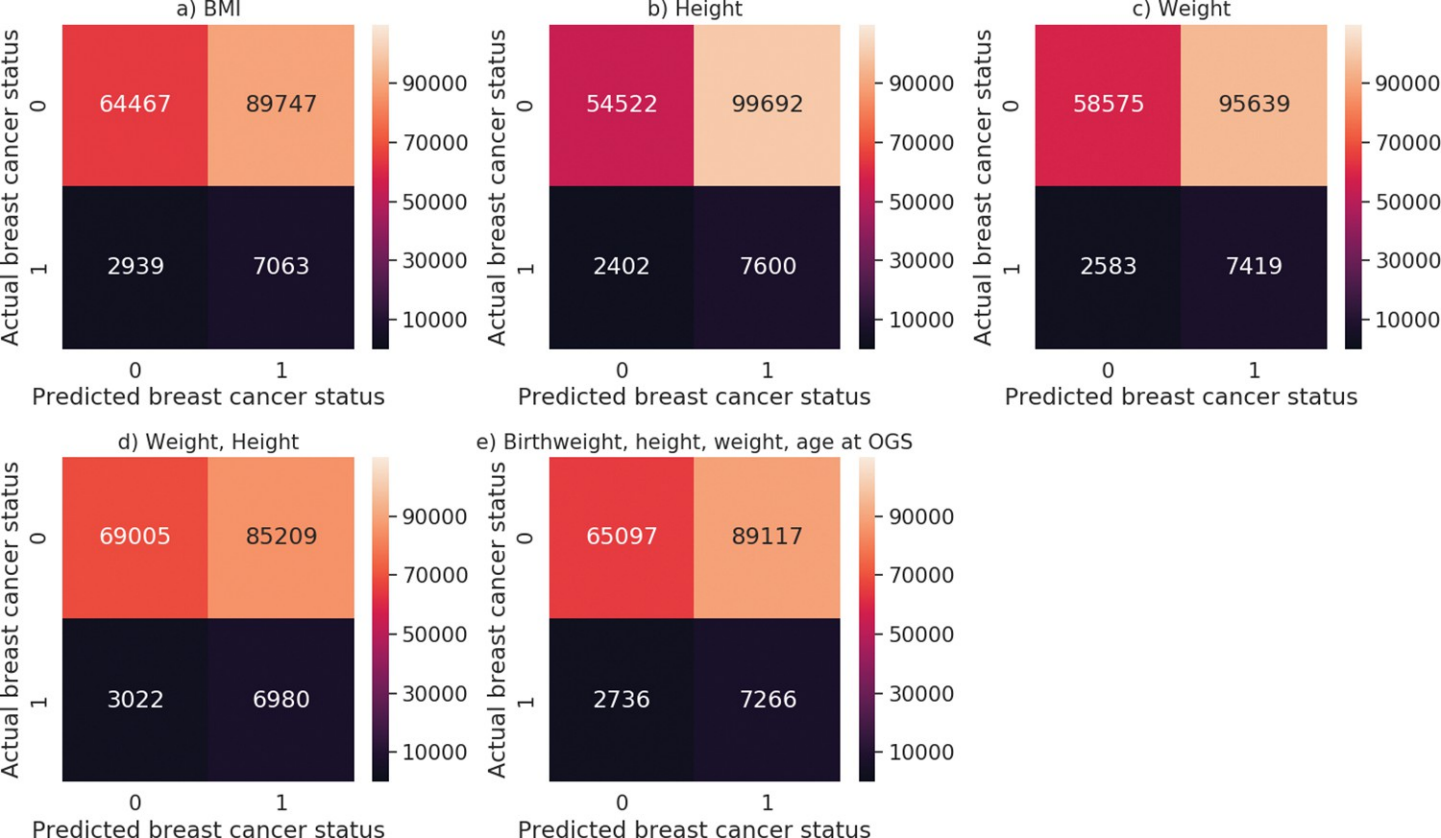
Confusion matrices for the neural networks. The panels show the network that included BMIs (Panel A), weights (Panel B), heights (Panel C), weights and heights (Panel D), and weights, heights, BW and age at the OGS (Panel E). 0 indicates those without breast cancer and 1 indicates those with breast cancer. Abbreviations: BW, birthweight; OGS, onset of the growth spurt.

The confusion matrices summarize the prediction results, with the rows showing the actual number of women with breast cancer and the columns the predicted number of breast cancer cases (Fig 3). The confusion matrix for the model including birthweight, weights, heights and age at OGS that yielded the highest AUC showed that among non-cancer cases, 89,117 (57.8%) women were predicted to develop breast cancer, and among breast cancer cases, 7266 (72.7%) women were predicted to develop breast cancer (Fig 3).

For comparison, the models were also examined using logistic regression. Using this method, as with the neural networks, the model including birthweight, heights, weights, and age at OGS yielded the highest AUC, while the model including only heights had the lowest AUC (Table 3). Compared to the neural networks, the logistic regression models yielded lower sensitivities, but higher specificities and accuracies. However, none of the logistic regression models predicted the risk of breast cancer with an accuracy above 0.510 (Table 3). Similar to the neural networks, the precision was low for all models (Table 3).

**Table 3:** Performance metrics for the logistic regression models predicting breast cancer*.

	Network performance metrics				
Input variables	AUC	Sensitivity	Specificity	Accuracy	Precision
BMI	0.560	0.58	0.51	0.51	0.07
Height	0.528	0.53	0.51	0.51	0.06
Weight	0.553	0.57	0.51	0.51	0.07
Height, weight	0.561	0.60	0.50	0.50	0.07
Birthweight, height, weight, age at OGS	0.564	0.60	0.50	0.50	0.07

* The input variables BMI, height, and weight includes values at each age between 7 and 13 years

Abbreviations: AUC, area under the curve; BMI, body mass index; OGS, onset of the growth spurt

## Discussion

Using neural networks, we found that the highest AUC was obtained from the network that was trained on birthweight, child heights, weights, and age at OGS, whereas the lowest AUC was obtained from the network with childhood heights as the single input. Nevertheless, the network that included childhood heights and weights as the input features had similar performance as the network with highest performance. As such, our results suggest that if breast cancer prediction models should account for early life body size, the inclusion of childhood heights and weights may be relevant.

Our neural networks yielded AUCs between 0.572–0.600. We acknowledge that these values within machine learning are considered as poor discrimination. Nonetheless, even though not directly comparable due to differences in methods, input features and data sources, the AUCs obtained in our study are similar to those reported in previous breast cancer prediction studies using traditional risk factors. One study evaluated the performance of the Rosner-Colditz breast cancer incidence model in two different datasets and compared the AUCs. The results showed that the AUC was 0.597 in the Nurse’s Health Study data and 0.589 in the California Teachers Study data [21]. When the authors compared the Gail model using these same data, AUCs of 0.562 and 0.547 were obtained from the Nurse’s Health Study data and the California Teachers Study data, respectively [21]. Our results are also comparable to those from a machine learning study using neural networks [22]. In this study, the authors reported an AUC of 0.608 from the best performing network, which was trained on a broad set of input variables including, among other factors, current age, ages at menarche and menopause, age at first live birth, BMI, HRT usage, number of first-degree relatives who had breast cancer, and race/ethnicity [22]. Interestingly, the highest AUC of 0.636 was reported in the study that updated the Rosner-Colditz incidence model by including adolescent somatotype [12]. However, in this model information on predicted percent mammographic density was also added, and the authors did not report the AUCs for the models when adding the two factors individually [12]. In comparison, another study examining the predictive value of adding mammographic density (percent dense area) to the Rosner-Colditz model reported AUCs of 0.619 and 0.659 among post-menopausal women not using HRT and post-menopausal women using HRT, respectively [23].

The low performance reported in our study and the previous studies likely reflects that although there are multiple identified risk factors for breast cancer, the majority of these are not strongly related to breast cancer risks. Nevertheless, the findings from a recent mendelian randomization study suggest that genetically predicted childhood body size at age 10 years (assessed as relative to peers) has an effect on breast cancer risk independent of adult body size [24]. Thus, these results indicate that early life is a window of susceptibility for breast cancer, which may explain why a model with childhood heights and weights performs similar to the models including several risk factors in adolescence and/or adulthood. The mechanisms underlying the associations between childhood height and weight/BMI and breast cancer, respectively, are not fully understood and likely differ. While childhood height may be linked to breast cancer through growth-regulating hormones, such as insulin-like growth factor-1 [25], lower breast density may mediate the association between excess childhood adiposity and breast cancer risk [26, 27].

We also used other parameters to evaluate the performance of the neural networks, and the sensitivities of our networks were moderately good, as at least 70% of the women with breast cancer were predicted correctly. However, the specificities were low and did not exceed 0.45. Thus, the networks incorrectly predicted at least 55% of women without breast cancer as being a breast cancer case. Similarly, the accuracies were below 0.50, which indicates that less than half of the women were predicted correctly as either cases or non-cases. A likely explanation for the higher sensitivity and lower specificity is that we forced the models to penalize misclassifications of the cancer cases more than misclassifications of non-cancer cases. Thus, the models overestimated the number of women with breast cancer, which resulted in the number of false positive exceeding the number of true negatives. Further, because of the imbalance between cancer and non-cancer cases, balanced weights were assigned to these two groups. As such, women from the false negative group were moved into the true positive group, but women from the true negative group were also moved to the false positive group. This may have impacted the precision of our networks, which did not exceed 0.075, meaning that only 7.5% of the women who were predicted as cancer cases actually developed cancer. As two of the aforementioned studies did not report performance metrics other than the AUCs, direct comparisons of our findings are precluded [12, 21]. However, the sensitivities and specificities of our networks are similar to those reported in the machine learning study [22], which reported a sensitivity of 0.599 and a specificity of 0.562 for its best performing network. Further, the precision of this network was 0.0287 [22], thus, indicating that even when a large number of adult factors are used for prediction, it was still very difficult to predict breast cancer accurately. Nevertheless, considering that we achieved corresponding performances with models trained on childhood heights and weights alone or in combination with other indices of early life body size, this suggests that breast cancer prediction models may benefit from including measures of childhood body size.

The strengths of our study include the large study population and the unique individual-level linkage with a nationwide database of breast cancer diagnoses [17]. In addition, validation of the DBCG database against the Danish Cancer Registry (established in 1942) [28] showed that the coverage of the DBCG database increased from 80% when it was initiated in 1977 to 95% in the mid-1990s [17]. Further, from 2006 onwards there has been complete agreement between the DBCG database and the National Pathology Registry (established in 1999) [29] on breast cancer status [30]. Thus, the validity of the breast cancer diagnoses during follow-up was high. It is also a strength of our study that childhood anthropometry was measured, which limits the potential for information bias associated with recall of childhood body size at later ages. Additionally, because the women were followed prospectively from childhood, this minimizes effects of survival bias until adult age for inclusion in this study. Due to the mandatory school health examinations and the universal health care system in Denmark, selection bias into the study population is limited. Another strength of the study is the method applied; machine learning techniques make it possible to analyze complex associations when compared to what is possible in traditional statistics, such as logistic regression models. Although the logistic regression analyses in our study yielded similar AUCs as the neural network analyses, this may be related to the structure of our data, rather than indicating that logistic regression analyses have equal performance as machine learning techniques. Our study also has limitations. As the coverage of breast cancer cases in the DBCG was 80% in the beginning of the study period, we cannot preclude some degree of misclassification of women with breast cancer as non-cases. Further, it is a limitation that we were unable to include information on other relevant risk factors used in other breast cancer prediction models such as ages at menarche and menopause, parity, and a family history of breast cancer. This could potentially have improved our networks, and thus, resulted in higher AUCs with better discrimination than those we obtained. Also, we were not able to distinguish between different subtypes of breast cancer, although this is relevant to consider since breast cancer is a heterogenous disease and the etiology likely differ by e.g., menopausal status and hormone receptor status. Additionally, we included repeated measurements of childhood height and weight, which are correlated, as input features and acknowledge that this might impact the generalizability of our results. Further, since these kinds of data are relatively rare, the possibilities for validating our networks in independent datasets may be limited. Nonetheless, future breast cancer prediction studies should aim at including measures of childhood heights and weights in order to obtain the predictive value of this relative to the traditional risk factors.

## Conclusion

We showed that neural networks trained and tested on measures of early life body size alone had relatively low performance in the prediction of breast cancer. Nevertheless, the performances of our networks were similar to those reported in other studies using traditional breast cancer risk factors. Further, we found that the best performing network was based on birthweight, childhood heights and weights as well as age at OGS as input features. However, this network had similar performance as the network including childhood heights and weights. There are multiple risk factors for breast cancer, but our findings suggest that there may be additional value in considering these measures in the prediction of breast cancer, despite the low performance found in our study.

## Supporting information

S1 Table Number and percentage of imputed values among women with and without breast cancer. Data are presented as n (%). Abbreviations: BMI, body mass index; OGS, onset of the growth spurt.
(DOCX)
