# Supplementary material for: Early life body size and puberty markers as predictors of breast cancer risk later in life: A neural network analysis
Source: PLoS One. 2024 Feb 9;19(2):e0296835. doi: 10.1371/journal.pone.0296835 (PMC10857724; doi:10.1371/journal.pone.0296835)
Supplement: S1 Table — Data are presented as n (%). Abbreviations: BMI, body mass index; OGS, onset of the growth spurt. (DOCX) [file pone.0296835.s001.docx]

**S1 Table**: Number and percentage of imputed values among women with and without breast cancer

|  | Breast cancer status | |
| --- | --- | --- |
| Input varibles | Yes (n = 10,002) | No (n = 154,214) |
| Birthweight, kg | 2863 (28.6) | 33,729 (21.9) |
| Height, m |  |  |
| Age 7 years | 475 (4.8) | 8400 (5.5) |
| Age 8 years | 264 (2.6) | 6600 (4.3) |
| Age 9 years | 194 (1.9) | 9320 (6.0) |
| Age 10 years | 196 (2.0) | 13,691 (8.9) |
| Age 11 years | 174 (1.7) | 14,696 (9.5) |
| Age 12 years | 264 (2.6) | 16,479 (10.7) |
| Age 13 years | 340 (3.4) | 20,514 (13.3) |
| Weight, kg |  |  |
| Age 7 years | 481 (4.8) | 8442 (5.5) |
| Age 8 years | 266 (2.7) | 6611 (4.3) |
| Age 9 years | 194 (1.5) | 9322 (6.0) |
| Age 10 years | 196 (2.0) | 13,695 (8.9) |
| Age 11 years | 174 (1.7) | 14,700 (9.5) |
| Age 12 years | 264 (2.6) | 16,479 (10.7) |
| Age 13 years | 340 (3.4) | 20,517 (13.3) |
| BMI, kg/m^2^ |  |  |
| Age 7 years | 481 (4.8) | 8442 (5.5) |
| Age 8 years | 266 (2.7) | 6611 (4.3) |
| Age 9 years | 194 (1.9) | 9321 (6.0) |
| Age 10 years | 196 (2.0) | 13,695 (8.9) |
| Age 11 years | 174 (1.7) | 14,700 (9.5) |
| Age 12 years | 264 (2.6) | 16,479 (10.7) |
| Age 13 years | 340 (3.4) | 20,513 (13.3) |
| Age at OGS, years | 3980 (39.8) | 83,876 (54.4) |

Data are presented as n (%)

Abbreviations: BMI, body mass index; OGS, onset of the growth spurt
